# Key role of phosphorylation sites in ATPase domain and Linker region of MLH1 for DNA binding and functionality of MutLα

**DOI:** 10.1038/s41598-023-39750-x

**Published:** 2023-08-02

**Authors:** May-Britt Firnau, Guido Plotz, Stefan Zeuzem, Angela Brieger

**Affiliations:** Goethe University Frankfurt, University Hospital, Medical Clinic 1, Biomedical Research Laboratory, Theodor-Stern-Kai 7, 60590 Frankfurt, Germany

**Keywords:** Cell biology, Molecular biology

## Abstract

MutLα is essential for human DNA mismatch repair (MMR). It harbors a latent endonuclease, is responsible for recruitment of process associated proteins and is relevant for strand discrimination. Recently, we demonstrated that the MMR function of MutLα is regulated by phosphorylation of MLH1 at serine (S) 477. In the current study, we focused on S87 located in the ATPase domain of MLH1 and on S446, S456 and S477 located in its linker region. We analysed the phosphorylation-dependent impact of these amino acids on DNA binding, MMR ability and thermal stability of MutLα. We were able to demonstrate that phosphorylation at S87 of MLH1 inhibits DNA binding of MutLα. In addition, we detected that its MMR function seems to be regulated predominantly via phosphorylation of serines in the linker domain, which are also partially involved in the regulation of DNA binding. Furthermore, we found that the thermal stability of MutLα decreased in relation to its phosphorylation status implying that complete phosphorylation might lead to instability and degradation of MLH1. In summary, we showed here, for the first time, a phosphorylation-dependent regulation of DNA binding of MutLα and hypothesized that this might significantly impact its functional regulation during MMR in vivo.

## Introduction

Different DNA repair mechanisms are responsible for the maintenance of the DNA information and are required to safeguard cellular functions and identities in all organisms. The major role of the DNA mismatch repair (MMR) is the correction of base–base mismatches and insertion-deletion loops, which occur during replication. In humans, the mismatch recognition can be initiated by two heterodimeric MutS complexes: The MSH2/MSH6 (MutSα) complex is primarily responsible for recognition of single base–base and small insertion deletion loops; the MSH2/MSH3 (MutSβ) complex is mainly responsible for the recognition of insertion-deletion loops containing up to 16 additional nucleotides in one strand^1,2^. After MMR initiation, the MLH1/PMS2 (MutLα) complex interacts with and binds to the MutS complex. MutLα plays a very important role in 5' as well as in 3' directed MMR, has an endonuclease function^3–6^, and is required for the recruitment of several other proteins involved in further steps of the process^2,7^.

Deficiency of MMR results in a massive increase of the mutation rate in affected cells and is especially associated with the development of colorectal cancer (CRC), where loss of MMR plays a role in up to one fifth of cases^8^. However, MMR deficiency is also frequently involved in other types of cancer^9^.

In sporadic CRCs, loss of MMR is detected in 12–17% of cases and is due to hypermethylation of the MLH1 promoter, which avoids *MLH1* gene transcription^10^. In the hereditary Lynch syndrome, which accounts for approximately 3% to *5*% of all CRC cases, germline mutations in the MLH1 gene are detectable in around 50% of patients whereas one-third of the detected alterations are non-synonymous, non-truncating variants, or missense variants, in the coding region^11,12^. In up to 3% of patients with Lynch syndrome, constitutional hypermethylation of the MLH1 promoter has been described, especially in patients with unclear family history^13^.

In addition to the lack of MLH1 caused by promoter hypermethylation or gene mutation, MLH1 function can also be limited by post-translational modifications. We previously showed that MLH1 can be phosphorylated by casein kinase 2 α (CK2α) at amino acid position serine 477 (S477) and we identified that phosphorylation of MLH1 at position S477 can switch off MMR activity in vitro^14^. Very recently, we were able to demonstrate in vivo, that CK2α is frequently overexpressed in CRCs and that a high nuclear/cytoplasmic expression of CK2α is responsible for enhanced phosphorylation of MLH1 and increased tumor mutation rates in these tumors^15^. Moreover, affected patients demonstrated a significantly reduced 5-year survival outcome^15^. Thus, we were able to show very clearly that post-translational modification of MLH1 by phosphorylation can also cause a loss or at least a marked restriction of MMR function, a condition that significantly drives tumor progression.

Furthermore, by mass spectrometry of MLH1, we were able to detect one serine located in the ATPase domain, serine 87 (S87), and two serines located in the linker region, serine 446 (S446) and serine 456 (S456), which were phosphorylatable in MLH1, albeit to a lesser extent^14^. The relevance of phosphorylation of MLH1 at S87, S446 and S456 is completely unknown so far. Therefore, the current study, we analyzed the influence of MLH1 phosphorylation at amino acid position S87, S446, S456 alone and in combination with S477 on the expression level, the DNA binding ability and the repair efficiency of MutLα.

## Materials and methods

### Cells

HEK293T cells, obtained from Dr. Kurt Ballmer (Paul Scherrer Institute, Villigen, Switzerland), were grown in DMEM with 10% Fetal Calf Serum and 1% Penicillin/Streptomycin. As previously described, MLH1 is not expressed in HEK293T^16^.

### Antibodies

Anti-MLH1 (G168-728) and anti-PMS2 (A16-4) were purchased from Pharmingen (BD Biosciences, Heidelberg, Germany). Anti-beta Actin (Clone AC-15) was from Sigma-Aldrich (Munich, Germany). Anti-phospho-AKT-substrate (23C8D2), used for the detection of phosphorylation of MLH1 at amino acid position serine 477 and hereinafter referred to as anti-p-MLH1, was obtained from Cell Signaling (New England Biolabs GmbH, Frankfurt, Germany).

Anti-fluorescence-labeled anti-mouse IRDye680LT and anti-fluorescence-labeled anti-rabbit IRDye800CW were from LI-COR (LI-COR Biosciences GmbH, Bad Homburg, Germany).

### Plasmids

The pcDNA3.1 + /PMS2 expression plasmid was described earlier^17^. The pEBG-2 T/MLH1 vector was generated by cloning the cDNA of MLH1 from the pcDNA3.1 + plasmid into the pEBG-2 T vector (a gift from David Baltimore (Addgene plasmid # 22227; http://n2t.net/addgene:22227; RRID:Addgene_22227;^18^) via the BamHI restriction site. This placed MLH1 downstream of the coding sequence for Glutathione-S-Transferase (GST).

Further pEBG-2 T plasmids containing MLH1-variants (MLH1^S87A^, MLH1^S446A^, MLH1^S456A^, MLH1^S87A/S477A^, MLH1^S446A/S477A^ and MLH1^S456A/S477A^) were subsequently prepared by site-directed mutagenesis using appropriate primers (Table 1) with the Q5 Site-Directed Mutagenesis Kit (New England Biolabs GmbH, Frankfurt, Germany). All resulting plasmids were confirmed by direct sequencing.

**Table 1:** Primers used for site directed mutagenesis.

MLH1 variant	Forward primer	Reverse primer
MLH1 S477A	5′- CATCGGGAAGATGCTGATGTGGAAATGG-3′	5′- CCATTTCCACATCAGCATCTTCCCGATG -3′
MLH1 S87A	5′- ACTAGTAAACTGCAGGCCTTTGAGGATTTAGCCA -3′	5′- TGGCTAAATCCTCAAAGGCCTGCAGTTTACTAGT -3′
MLH1 S446A	5′- GCTGCCAAAAATCAGGCCTTGGAGGGGGATACAA -3′	5′- TTGTATCCCCCTCCAAGGCCTGATTTTTGGCAGC -3′
MLH1 S456A	5′- ACAACAAAGGGGACTGCCGAAATGTCAGAGAAGA -3′	5′- TCTTCTCTGACATTTCGGCAGTCCCCTTTGTTGT -3′

The pEGFP_C1 plasmid, negative control plasmid for transfection control and negative control for MMR assay, was purchased from Clontech Laboratories (Clontech Laboratories, Mountain View, United States of America).

The pEBG-2 T empty vector containing only GST was a used as a negative control plasmid for the electrophoretic mobility shift assay (EMSA).

### Cell transfection and treatment

HEK293T at 50–70% confluence were transiently a) co-transfected with pEGB-2 T/MLH1 and pcDNA3.1 + /PMS2 as positive controls, b) co-transfected with pEGB-2 T constructs containing different MLH1 variants (pEGB-2 T/MLH1^S87A^, pEGB-2 T/MLH1^S446A^, pEGB-2 T/MLH1^S456A^, pEGB-2 T/MLH1^S477A^, pEGB-2 T/MLH1^S87A/S477A^, pEGB-2 T/MLH1^S446A/S477A^, pEGB-2 T/MLH1^S456A/S477A^) and pcDNA3.1 + /PMS2 and c) single transfected with pEGFP_C1 or empty pEGB-2 T as negative controls. For the generation of protein-extracts used in the MMR-assay, transfection of HEK293T cells was carried out in ø100mm dishes using 5 µg plasmid DNA and 2 µL/mL of the cationic polymer polyethylenimine (PEI) (Polysciences, Warrington, PA, USA; stock solution 1 mg/mL). For immunoprecipitation experiments, transfection of HEK293T cells was performed in ø100mm dishes as well, 5 µg Plasmid, 2 µL/mL PEI. For generation and overexpression of GST-tagged MutLα, HEK293T cells were co-transfected in cell culture dishes (ø145mm) using 12.5 µg plasmid DNA and 4.375 µL/mL of PEI. At 48 h post-transfection, cells were directly harvested or treated 8 h with Calyculin (50 nM) or DMSO as a control if indicated.

Finally, cells were harvested, protein extracts were generated and used for immunoprecipitation and analyzed by Western blotting or used as required. All experiments were performed at least three times.

### Protein extraction

Denatured protein extracts of transiently transfected HEK293T cells, used for Western blotting, were prepared as described earlier^15^. In brief, cells were isolated by re-suspending the cells directly in medium and centrifuging (3000 g, 3 min, 4 °C). The cells were washed in 1 mL PBS and centrifuged again (3000 g, 3 min, 4 °C). The supernatant was discarded and cells were solved in lysis reagent (CelLytic™ M Lysispuffer Sigma-Aldrich (St. Louis, Missouri, USA)) combined with cOmplete™ Protease Inhibitor Cocktail Roche (Roche Pharma, Basel, Switzerland)), incubated on ice for 5 min, lysed by ultrasound for 10 s at 35% power, and centrifuged (14,000 *g*, 10 min, 4 °C). The supernatant (whole protein extract) was transferred to a new Eppendorf tube and stored at −20°C until use.

Whole cell extracts used for the MMR assay were generated as described before^19^. In brief, native protein extracts were generated by re-suspending PBS-washed cells in 2 times their packed cell volume of ice-cold hypotonic buffer (10 mM HEPES (pH 7.6), 5 mM MgCl2, 10 mM NaCl, 10 mM NaF, 0.1 mM EDTA, supplemented with 0.2 mM PMSF, 0.5 mM DTT directly before use). This suspension was carefully homogenized, incubated on ice for 5 min and snap-frozen in liquid nitrogen for 2 min for lysis. Afterwards, the cell suspension was thawed on ice and after 1 h, an identical volume of hypertonic buffer (10 mM HEPES (pH 7.6), 5 mM MgCl2, 830 mM NaCl, 10 mM NaF, 0.1 mM EDTA, 34% (v/v) glycerol, supplemented with 0.2 mM PMSF and 0.5 mM DTT directly before use) was added. The suspension was rocked on ice for 30 min with gentle agitation and centrifuged (14,000g, 10 min, 4 °C). The supernatant (native cell extract) was transferred to a new Eppendorf tube. The protein concentration was determined according to Bradford before the samples were frozen in liquid nitrogen and stored in aliquots at −80°C until further use.

### Generation of nuclear extracts

10 cell culture dishes (ø145mm) of untransfected HEK293T cells were harvested, washed with PBS, pelleted and re-suspended in three times their packed cell volume of ice-cold hypotonic buffer (20 mM HEPES pH 7.6, 5 mM KCl, 0.5 mM MgCl_2_, supplemented with 0.2 mM PMSF, 0.5 mM DTT directly before use) and lysed with Dounce pestle B until lysis was sufficient. After centrifugation (10,000 g, 2 min, 4 °C), the cytoplasmic supernatant was again removed and centrifuged (12,000 g, 3 min, 4 °C) for removal of all cytoplasmic residuals. The pellet was re-suspended in 0.4 times the pellet volume of re-suspension-buffer (20 mM HEPES–KOH pH 7.6, 10% sucrose, supplemented with 0.2 mM PMSF and 1 mM DTT directly before use) with cOmplete™ Protease Inhibitor Cocktail Roche (Roche Pharma, Basel, Switzerland) and 0.4 times the pellet volume of high-salt buffer (50 mM HEPES–KOH pH 7.6, 10% sucrose, 840 mM KCl) was added under agitation. Extraction was performed for 30 min and extracted nuclei were removed by centrifugation (21,000 g, 30 min, 4 °C). Finally, the supernatant was dialyzed 3 h against 100 times the volume of dialysis buffer (25 mM HEPES pH 7.6, 100 mM KCl, 0.1 mM EDTA, supplemented with 0.2 mM PMSF and 0.5 mM DTT directly before use). The extract was centrifuged (21,000 g, 10 min, 4 °C), snap-frozen in liquid nitrogen and stored in aliquots at −80 °C.

### Immunoprecipitation

Immunoprecipitations were carried out as described in Wessbecher et al.^14^ using 500 µg of whole cell extract from MutLα wild type (wt) or MutLα variant overexpressing HEK293T cells in a total volume of 1000 µl precipitation buffer (50 mM HEPES–KOH (pH 7.6), 100 mM NaCl, 0.5 mM EDTA, 0.2 mM PMSF, 0.5 mM DTT, 1% Triton X-100) with 2 µg of anti-MLH1 (G168-728). After one hour of agitated incubation at 4 °C, 20 µl protein G sepharose (Santa Cruz Biotechnology, Heidelberg, Germany) were added and incubation continued for 3 h. Precipitates were extensively washed in cold precipitation buffer using SigmaPrep™ spin columns (Sigma, Munich, Germany). Success of washing was always confirmed by running samples without antibody in parallel. The SpinColumns were boiled in SDS-PAGE sample buffer for 5 min, followed by centrifugation (10,000 g, 1 min) and proteins were separated on 10% polyacrylamide gels, followed by Western blotting on nitrocellulose membranes and antibody detection using standard procedures.

### Western blotting

Proteins were separated on 10% polyacrylamide gels, followed by Western blotting on nitrocellulose membranes and antibody detection using standard procedures. Nitrocellulose membranes on which immunoprecipitated proteins were blotted were cut between 50 and 75 kDa, only the upper part of the membrane was incubated with antibody. This prevented hybridisation of the antibody of interest to the antibody used for precipitation.

Fluorescent-labeled secondary antibodies (anti-mouse 680 LT from LiCor Bioscience, anti-mouse 800 CW from LiCor Bioscience, anti-rabbit 680 LT from LiCor Bioscience) were used to detect signals in a FLA-9000 scanner (Fujifilm, Tokyo, Japan). If indicated, the band intensity of the protein expression was quantified using the Multi Gauge V3.2 program (Fujifilm, Tokyo, Japan).

The amount of p-MLH1^S477^ was detected after immunoprecipitation and quantified in correlation to total MLH1 levels as previously described^14^ using Multi Gauge V3.2. p-MLH1^S477^ levels were calculated in relation to MLH1 set to 100%. Thereafter, all p-MLH1^S477^ level were normalized to the p-MLH1^S477^ level of the MLH1 wt. All experiments were performed at least three times.

### Generation of GST-tagged recombinant MutLα

GST-MLH1 and GST-MLH1 variants were coexpressed with PMS2 wt in 7 cell culture dishes (ø145 mm) of HEK293T cells as detailed above. Cells were harvested after 48 h in lysis buffer (50 mM Trizma pH 7.4, 0.27 M Sucrose, 1 mM Na-Ortho-Vanadate, 1 mM EDTA, 1 mM EGTA,10 mM Na-ß-glycerolphosphate, 50 mM NaF, 5 mM Na-pyrophosphate, 1% Triton-x-100) and centrifuged (4000 rpm, 15 min, 4 °C). The Supernatant was incubated (4 °C) with 0.5 ml Glutathione-sepharose 4B (GE Healthcare, Chicago, U.S.A) pre-washed twice with one volume of buffer A (50 mM Trizma pH 7.4, 0.1 mM EGTA, 0.1% β-Mercaptoethanol) and once with one volume of lysis buffer. After 2 h, the Glutathione-sepharose-suspension was washed 4 times with 10 ml lysis buffer with 500 mM NaCl, 8 times with 10 ml buffer A, and once with 10 ml buffer A plus 0.26 M Sucrose. Thereafter, 500 µl buffer A plus 0.26 M Sucrose and 80 mM glutathione was added per 500 µl Glutathione-sepharose and this suspension was incubated again for 2 h. Finally, the Glutathione-sepharose was pelleted (2500 rpm, 2 min, 4 °C) and used for EMSA, since a large amount of GST-tagged MutLα was still bound. In addition, the supernatant was loaded on SigmaPrep columns (Sigma Aldrich, Munich, Germany) and GST-tagged MutLα was eluted by centrifugation (3000 rpm, 5 min, 4 °C). The elution was repeated with 250 µl buffer A plus 0.26 M Sucrose and 80 mM glutathione. Recombinant GST-tagged MutLα was frozen with liquid nitrogen and stored at -80 °C until use.

### Synthesis of GT-DNA-substrate

Generation of GT-DNA-substrate was carried out using pUC19 CPDC and pUC19 CPD^20^ plasmid-constructs on which the MMR assay of this manuscript is based on. Both vectors only differ in one base, which enabled us to generate a suitable DNA substrate for the EMSA: a double stranded DNA fragment carrying a G/T mismatch. First, amplification of the 101 bp pUC19 CPDC-depending fragment was performed using 100 ng pUC19 CPDC vector together with a 5′-P-tagged forward primer and a 5′-Atto680-tagged reverse primer (Table 2), the pUC19 CPD -depending fragment was amplified using 100 ng of pUC19 CPD together with a 5′-Atto680-tagged forward primer and a 5′-P-tagged reverse primer (Table 2). 10 µl of PCR fragments were treated with 0.25U of exonuclease λ (M0262S (5U/µl), New England Biolabs GmbH, Frankfurt, Germany) for 10 min at 37 °C followed by adding further 0.5U exonuclease λ and incubating again for 10 min at 37 °C to catalyze the removal of nucleotides from the 5’-phosphorylated double-stranded DNA, respectively. Resulting single stranded DNA fragments were purified, mixed 1/1, boiled at 95 °C for 5 min, and cooled down at room temperature. The mixture was centrifuged (14,000 rpm, 5 min, RT) and the supernatant was transferred to a new test tube. 10 µl of this supernatant were digested with 5U of exonuclease I (M0293S (20U/µl), New England Biolabs GmbH, Frankfurt, Germany) and incubated for 1 h at 37 °C to remove residual abundant nucleotides from linear single-stranded DNA in the 3' to 5' direction. Finally, the GT-DNA substrate was precipitated with sodium acetate 3 M and ethanol, washed with 70% ethanol and re-suspended in H_2_O.

**Table 2:** Primers used for generation of GT-DNA-substrate.

Used plasmid	Forward primer	Reverse primer
pUC19 CPDC^20^	5′-P-ACATTTCCCCGAAAAGTGCC-3′	5′-Atto680-AGCAAGGCAGTGAGCGAGGA-3′
pUC19 CPD^19^	5′-Atto680-ACATTTCCCCGAAAAGTGCC-3′	5′-P-AGCAAGGCAGTGAGCGAGGA-3′

### Electrophoretic mobility shift assay

To analyze binding of GST-tagged MutLα wt and variants to DNA, a) 10 µl of Glutathione-sepharose-bound protein samples or b) 10 µl dephosphorylated Glutathione-sepharose-bound protein samples pretreated with 10 units of CIP (at 37 °C for 1 h), were first combined with 1.5 µl 10 mM ATP in 20 mM HEPES pH 7.5, 40 µg/ml BSA, 1 mM DTT, 100 mM NaCl, mixed with 95 ng GT-DNA-substrate and incubated on ice. After 5 min, 1 µl of 8% (w/v) sucrose and 2 µl of 75% glycerol were added to each sample. Now, the electrophoretic mobility shift assay (EMSA) was carried out in 4% non-denaturing polyacrylamide gels. After a first gel run for 30 min (150 V with ice-cold 0.5xTBE-buffer (pH 7.5)) without the addition of any probe, samples were applied and electrophoresed for 2 h at 48 V (in a dark room). Thereafter, gel-included glass plates were placed in a plastic bag and GT-DNA-substrate detection was performed at 685 nm using a FLA-9000 scanner (Fujifilm, Tokyo, Japan) and quantified using the Multi Gauge V3.2 program (Fujifilm, Tokyo, Japan). Subsequently, the DNA bound protein-amount was determined via Western blotting and compared to a known concentration of a control sample analyzed in parallel. All experiments were performed at least three times.

### Analysis of MMR activity

The analysis of the MMR activity of the MutLα variants was determined in vitro as described^19,21^. Briefly, 2.5 μg protein extracts of transfected HEK293T cells were mixed with 50 μg nuclear extract of HEK293T cells and 35 ng of DNA substrate containing an intact AseI restriction site, a G-T mismatch in between an (not digestible) EcoRV restriction site and a 3′ single-strand nick at a distance of 83 bp to guide the MMR direction. If the tested heterodimer is fully qualified for mismatch repair, then the G-T mismatch will be corrected and the EcoRV restriction site will be restored. After incubation at 37 °C (20 min) the reaction was stopped, following by purification and digestion of the DNA substrate with AseI (5 units/15 µl) and EcoRV (10 units/15 µl). The restriction fragments were separated in agarose gels, analyzed using GelDoc XR plus detection and band intensities were quantified using Image Lab version 3.0 (Bio-Rad). If the substrate is efficiently repaired the AseI and EcoRV digestion will result in three bands: a 2.0 kb band corresponding to singly AseI cut (uncorrected and in excess added) DNA substrate, a 1.2 kb and a 0.8 kb band caused by the successfully corrected and restricted EcoRV restriction site. The repair efficiency (e) was calculated as: (e) = intensity of bands of repaired substrate/intensity of all bands of substrate. This result is independent of the amount of DNA recovered through plasmid purification. The (e) of MutLα variants was analyzed in direct comparison with MutLα wt that had been produced in parallel and calculated as (e)_relative_ = (e)_variant_/(e)_wild-type_ × 100.

In addition, it should be mentioned that a small amount of Calyculin is abundant in the MMR assay during the protocol when adding whole protein extracts of Calyculin treated cells. To exclude that this amount of Calyculin has any influence, an additional control experiment was performed in the current study. The amount of Calyculin present in the protein extract was calculated and was added separately and co-incubated according to the MMR assay protocol. In brief, 2.5 μg protein extracts of untreated MutLα wt expressing HEK293T cells, 50 μg nuclear extract of HEK293T cells, 35 ng of DNA substrate and 0.815 nM Calyculin were mixed and incubated. In parallel, the same reaction mixture without the addition of Calyculin was prepared and incubated. The repair efficacy of assays with Calyculin supplementation was compared to the efficacy of assays without Calyculin. All experiments were performed at least three times.

### Nano differential scanning fluorimetry

Protein stabilities of recombinant GST-tagged MutLα variants were compared to the protein stability of recombinant GST-tagged MutLα wt as previously described^22^ on the basis of thermal stability, using nano-differential scanning fluorimetry (nanoDSF), a modified version of differential scanning fluorimetry, working with a Prometheus NT.48 (NanoTemper) with standard capillaries (NanoTemper).

This label-free technique uses the intrinsic fluorescence of the aromatic amino acids tryptophan and tyrosine to determine protein folding and stability. The fluorescence is excited at 280 nm and is detected at 330 nm and 350 nm. nanoDSF determines the apparent melting temperature (T_m_), where half of the protein is unfolded, and onset temperature (T_onset_) of thermal unfolding by measuring the ratio of the fluorescence intensity at 330 nm and 350 nm as a function of temperature. The derivation of F350/330 is used for data analysis. The protein samples were set up to a concentration of 1.2 mg/mL for every measurement, loaded into capillaries by capillary force action and placed into the instrument. The capillaries were heated from 20 to 80 °C with a heating rate of 2 °C /min and the changes in the fluorescence ratio (F350/F330) were monitored. For each experiment, a capillary containing only buffer A (50 mM Trizma pH 7.4, 0.1 mM EGTA, 0.1% β-Mercaptoethanol) plus 0.26 M Sucrose and 80 mM glutathione was used to calculate the blank value.

### Statistical analysis

Unpaired two-tailed T-tests for normality, followed by Welch correction for uneven variations, and the Mann–Whitney-U test for non-normally distributed data were used to assess statistical significance.

All calculations were analyzed using the software GraphPad Prism 7 for Windows, Version 7.04 (La Jolla, CA, USA). The data shown are means ± SD, unless otherwise stated, the following p-values were considered as statistically significant: * p < 0.05, ** p < 0.005, *** p < 0.005, **** p < 0.0001 unless otherwise stated.

## Results

### Phosphorylation at amino acid positions S87, S446 or S456 has no influence on phosphorylatability of MLH1 at position S477

**Figure 1:**
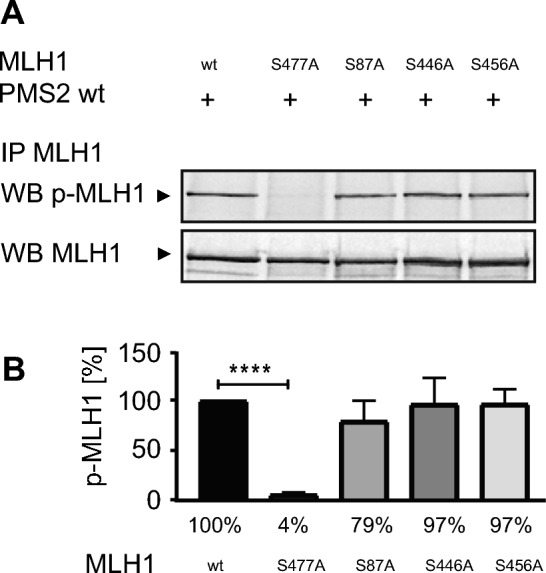
Amino acid positions S87, S446 or S456 have no influence on phosphorylatability of position S477. Using immunoprecipitated MutLα and a specific antibody which recognizes the phospho-S477-motif of MLH1 (**A**) the expression of p-MLH1^S477^ was determined in MutLα wt as well as in MLH1^S477A^/PMS2, MLH1^S87A^/PMS2, MLH1^S446A^/PMS2, MLH1^S456A^/PMS2 variant overexpressing HEK293T cells (shown as cropped blots) and (**B**) expression levels of five independent experiments were quantified (mean ± S.D.) using Multi Gauge V3.2 program and normalized in relation to the value of MLH1 wt levels. p-MLH1^S477^ was well detectable in all protein extracts despite in the extract of HEK293T cells overexpressing the non-phosphorylatable MLH1^S477A^/PMS2 variant. P-values were calculated by paired T-test. **** p < 0.0001; n = 5.

We previously detected that MLH1 harbors three additional amino acids, besides S477, that can be phosphorylated. Since it is known that different phosphorylation sites within one protein can influence each other, we first analyzed if phosphorylation of one of these amino acid positions (S87, S446 and S456) impacts the phosphorylatability of MLH1 at position S477. Therefore, non-phosphorylatable MLH1 variants (MLH1^S87A^, MLH1^S446A^, MLH1^S456A^, and MLH1^S477A^) were co-expressed together with PMS2 wt protein in HEK293T cells for 48 h. To stabilize phosphorylated amino acids, cells were then treated with Calyculin for 8 h, whole cell extracts were isolated, MutLα was immunoprecipitated using anti-MLH1 and the amount of p-MLH1^S477^ as well as PMS2 was compared via Western blotting (Fig. 1A, Figure S1). Quantification of bands was performed by using Multi Gauge V3.2 and correlated to those of MLH1 wt expressing cells which were set to 100% (Fig. 1B).

We detected that the phosphorylatability of S87, S446 and S456 did not affect the amount of p-MLH1^S477^ (Fig. 1A + B). The amount of p-MLH1^S477^ was comparable in the MLH1^S87A^/PMS2, the MLH1^S446A^/PMS2, as well as in the MLH1^S456A^/PMS2 variant after Calyculin treatment and similar to the amount of p-MLH1^S477^ of expressed MutLα wt. MLH1^S87A^/PMS2 showed 79% ± 21%, MLH1^S446A^/PMS2 showed 96% ± 26% and MLH1^S456A^/PMS2 showed 96% ± 15% of the p-MLH1^S477^ amount precipitated from MutLα wt extracts. No p-MLH1^S477^ could be detected at position S477 using the non-phosphorylatable MLH1^S477A^ /PMS2 variant (4% ± 2%) (Fig. 1B).

### Amino acid changes at S87, S446, S456 and S477 do not affect the amount of whole MLH1 expression

**Figure 2:**
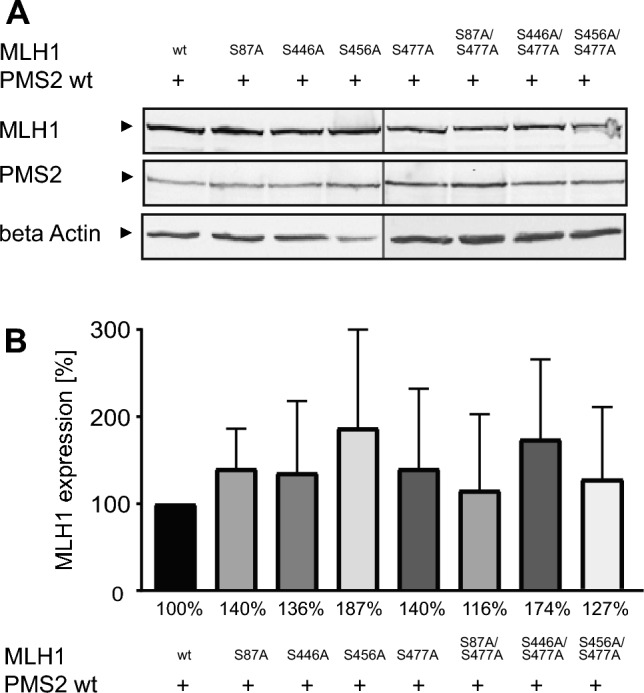
Changes from serine to alanine in phosphorylatable positions of MLH1 have no significant impact on the expression level of MutLα. HEK293T cells were cotransfected with pEBG-2 T/MLH1 or different pEBG-2 T/MLH1 variants and pcDNA3.1 + /PMS2. Protein expression of MLH1 and PMS2 was (**A**) analyzed via Western blotting (shown as cropped blots) and (**B**) expression level of MLH1 was quantified using Multi Gauge V3.2 program and normalized in relation to the value of MLH1 wt levels. The expression levels of all MLH1 variants were similar compared to MLH1 wt as well as all corresponding PMS2 levels. The shown percentages are given as rounded numbers in the figure. n = 3.

To investigate the impact of amino acid changes from serine to alanine at positions S87, S446, S456 and S477 of MLH1 on the amount of protein expression of MutLα, HEK293T cells were transiently cotransfected with pEBG-2 T/MLH1 (or the respective MLH1 variant) and pcDNA3.1 + /PMS2 and the amount of MLH1 and PMS2 for each variant was determined via Western blotting. As shown in Fig. 2 (and Figure S2), MLH1 as well as PMS2 expression of all MutLα variants was slightly increased, but in general very similar compared to the MLH1 and PMS2 expression of MutLα wt and showed no significant differences. In detail, the relative MLH1 expression level of MLH1^S87A^/PMS2 was 140.5% ± 45.58%, of MLH1^S446A^/PMS2 it was 136% ± 81.89%, of MLH1^S456A^/PMS2 it was 187.3% ± 112.8%, of MLH1^S477A^/PMS2 it was 140.6% ± 91.42%, of MLH1^S87A/S477A^/PMS2 it was 115.8% ± 86.86%, of MLH1^S446A/S477A^/PMS2 it was 174.4% ± 91.35% and of MLH1^S456A/S477A^/PMS2 it was 126.7% ± 82.27%. Thus, the amino acid changes seem to have no influence on the expression level of MLH1 or PMS2.

### Amino acid position S87 is of great importance for DNA binding of MutLα

**Figure 3:**
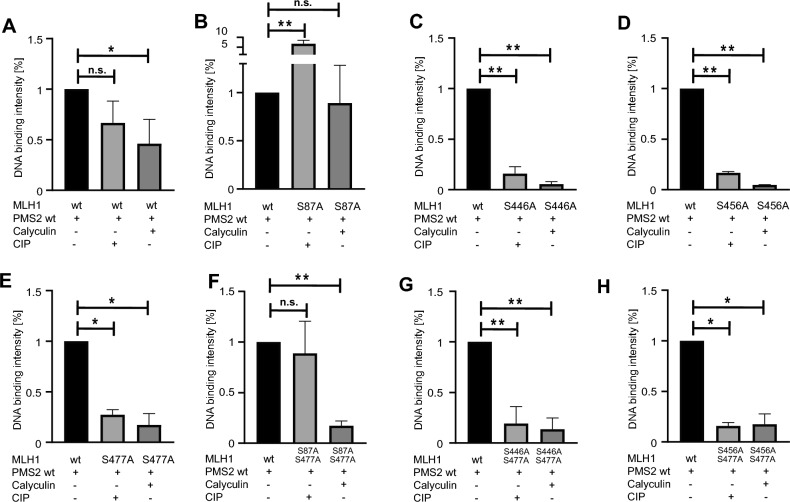
Phosphorylation of MLH1 inhibits DNA binding of MutLα. The relevance of phosphorylation of MLH1 was determined by using EMSA. The assay was performed using a missense mutation harboring fluorescence labeled DNA substrate and different GST-tagged MutLα variants. The DNA binding of non-phosphorylated (untreated) or phosphorylated (Calyculin treated) MutLα variants was compared to the DNA binding of MutLα wt, respectively. Shown are the results of (**A)** MutLα wt, (**B**) MLH1^S87A^/PMS2, (**C**) MLH1^S446A^/PMS2, (**D**) MLH1^S456A^/PMS2, (**E)** MLH1^S477A^/PMS2, (**F**) MLH1^S87A/S477A^/PMS2, (**G**) MLH1^S446A/S477A^/PMS2 and (**H**) MLH1^S456A/S477A^/PMS2. P-values were calculated by Mann–Whitney-U test. * p < 0.05; ** p < 0.005; n.s. = not significant; n = 3–5.

MutLα has been shown to be able to bind to the DNA, which is required, for example, for its endonuclease activity during the MMR^3–6^. In order to determine the influence of phosphorylation of MLH1 at position S87, S446, S456 and S477 on DNA binding of MutLα an EMSA was performed. For this assay fluorescently labeled double-stranded DNA with a mismatch, recombinant GST tagged MutLα wt and MutLα variants were used. Since the amount of protein bound to the DNA was the same for purified GST-tagged MutLα in comparison to Glutathione-sepharose-bound MutLα (Figure S3A), Glutathione-sepharose-bound protein samples were used for all EMSAs. The different recombinant MutLα variants were subjected to two treatments: Calf intestinal phosphatase (CIP), causing complete dephosphorylation, and treatment with the serine/threonine protein phosphatase inhibitor Calyculin, causing hyperphosphorylation. The untreated recombinant MutLα wt complex was used as a control in all experiments and, after calculating the exact amount of DNA-bound protein via Western blotting in parallel, the DNA-bound amount of MutLα wt was set to 100%, respectively (Fig. 3; Figure S3; Figure S4A-G).

Hyperphosphorylation of MutLα wt leads to significantly decreased DNA binding while dephosphorylated MutLα binds as similar as the untreated MutLα complex to the DNA (Fig. 3, upper panel, first graph from the left; Figure S3B).

Comparing the DNA binding intensity of MutLα wt with the respective MLH1/PMS2 variants, it becomes clear that phosphorylation of position S87 seems to play the most important role for DNA binding. The Calyculin treated, fully phosphorylated, but at position S87 non-phosphorylatable MLH1^S87A^/PMS2 variant shows no difference in DNA binding compared to the unphosphorylated MutLα wt (Fig. 3, upper panel, second graph from the left; Figure S4A). Interestingly, the completely dephosphorylated, CIP treated, MLH1^S87A^/PMS2 variant even binds significantly better to DNA than the MutLα wt.

In the case of the MLH1^S87A/S477A^/PMS2 variant, only the dephosphorylated form was able to bind to the DNA very well and in a comparable amount as the MutLα wt complex (Fig. 3, lower panel, second graph from the left; Figure S4E). In contrast, Calyculin-treatment, which leads to phosphorylation at position S446 and S456, inhibits binding of MLH1^S87A/S477A^/PMS2 variant and showed significantly less DNA bound protein (Fig. 3, lower panel, second graph from the left; Figure S4E). However, none of the tested variants bind to DNA with or without phosphorylated serines (Fig. 3, upper panel, third and fourth graph from the left; lower panel, first, third and fourth graph from the left; Figure S4B-D; Figure S4F + G).

## Phosphorylation of MLH1 prevents MMR

**Figure 4:**
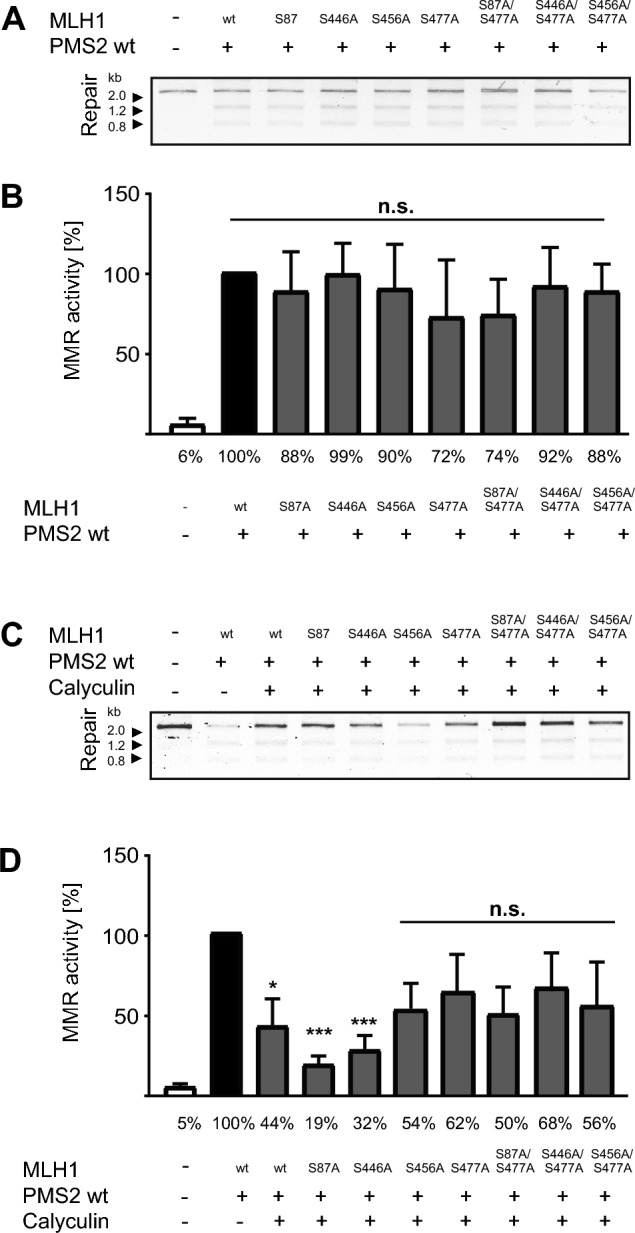
Phosphorylation levels of phosphorylatable amino acids of MLH1 significantly impact the MMR function of MutLα**.** An in vitro MMR assay was performed to determine the impact of phosphorylation at positions S87, S446, S456 or S477 on the functionality of MutLα. DNA MMR substrate was incubated with differently phosphorylatable MutLα variants and the MMR activity of the (**A + B**) non-phosphorylated (untreated) and the (**C + D**) phosphorylated (Calyculin treated) form of these variants was compared to the MMR activity of MutLα wt, repectively. All non-phosphorylated MutLα variants showed MMR activity similar to that of MutLα wt; significant differences were detectable using the phosphorylated forms of these proteins. The shown percentages are given as rounded numbers in the figure. P-values were calculated by unpaired two-tailed t-test, followed by Welch correction and the Mann–Whitney-U test. * p < 0.005; *** p < 0.0001; n.s. = not significant; n = 4–6.

To investigate the MMR relevance of MLH1 phosphorylation at amino acid positions S87, S446, S456 and S477, an in vitro MMR assay was performed. The analysis was carried out using protein extracts of untreated (Fig. 4A + B) and Calyculin treated (Fig. 4C + D) HEK293T cells which were transiently cotransfected with pEBG-2 T plasmids containing MLH1 wt or the different MLH1-variants and pcDNA3.1 + /PMS2 wt. Protein extract of transiently pEGFP_C1 transfected HEK293T served as a negative control.

Numerical values were quantified and set in relation to MutLα containing MLH1 wt (positive control) (100 ± 0). The results indicate that all non-phosphorylated MutLα variants were able to repair the DNA substrate in a similar manner as MutLα wt (Fig. 4A + B, Figure S5A). Variant MLH1^S87A^/PMS2 showed 88.33% ± 24.60% repair activity, MLH1^S446A^/PMS2 showed 98.98% ± 19.24% repair activity, MLH1^S456A^/PMS2 showed 89.93% ± 27.64% repair activity, MLH1^S477A^/PMS2 showed 72.37% ± 35.48% repair activity; MLH1^S87A/S477A^/PMS2 showed 73.91% ± 22.11% repair activity, MLH1^S446A/S477A^/PMS2 showed 91.57% ± 24.05% repair activity and MLH1^S456A/S477A^/PMS2 showed 88.38% ± 17.03% repair activity.

After Calyculin treatment and hyperphosphorylation of the used MutLα variants, a different picture has been drawn. Some phosphorylated protein-complexes showed significantly reduced MMR activity compared to the unphosphorylated MutLα wt while the MMR functionality of other MutLα variants was impaired but still present after Calyculin treatment (Fig. 4C+D, Figure S5B). The reduction of the MMR activity of phosphorylated MutLα wt was significant with 44.27% ± 20.02%, of the phosphorylated MLH1^S87A^/PMS2 the activity with 19.35% ± 3.91% was the lowest, and of phosphorylated MLH1^S446A^/PMS2 the MMR activity was also very weak and significantly reduced with 32.10% ± 3.68%. However, for all other variants no significant reduction in MMR activity could be determined. The MMR activity of phosphorylated MLH1^S456A^/PMS2 was 53.56% ± 16.78%, of phosphorylated MLH1^S477A^/PMS2 it was 61,98% ± 20.22%, of phosphorylated MLH1^S87A/S477A^/PMS2 it was 49.38% ± 12.64%, of phosphorylated MLH1^S446A/S477A^/PMS2 it was 67.82% ± 20.14% and of phosphorylated MLH1^S456A/S477A^/PMS2 it was 56,46% ± 27.02% in comparison to the untreated MutLα wt control (Fig. 4C + D, Figure S5B).The negative control performed with protein extracts of pEGFP_C1 transfected HEK293T cells demonstrated no MMR activity, with 4.70% ± 2.10% (Fig. 4A + B, Figure S5A) or 5.14% ± 2.80% (Fig. 4C + D, Figure S5B). The supplementation of Calyculin itself, separately added to the assay during incubation with the DNA substrate, was without influence on the MMR efficacy (Figure S6).

## Thermostability of MutLα is significantly influenced by the phosphorylation status of MLH1

**Figure 5:**
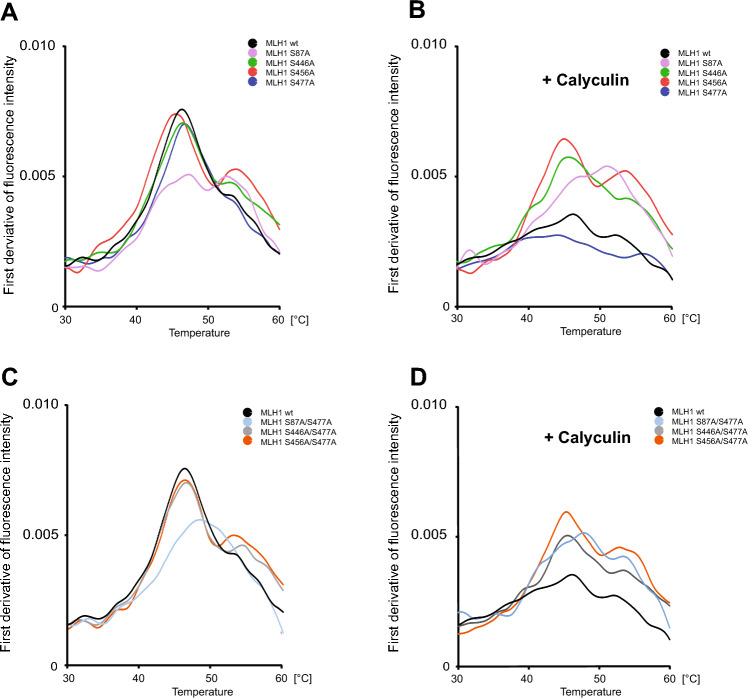
Phosphorylation status of MLH1 influences the thermostability of MutLα. The thermostability of recombinant, unphosphorylated and phosphorylated versions of MutLα wt and different MutLα variants was determined using nano-differential scanning fluorimetry (nanoDSF). Measurements of (**A + C**) unphosphorylated and (**B + D**) phosphorylated proteins were performed and data are shown as the first derivative of the fluorescence intensity (Fi) (Fi350nm/Fi330nm).

The influence of phosphorylation of MLH1 at positions S87, S446, S456 and S477 of MLH1 on the general protein stability was determined by nanoDSF using recombinant purified MutLα wt and the different MutLα variants (Fig. 5). As demonstrated in Fig. 5 A + C, all untreated MutLα variants, except those carrying the non-phosphorylatable amino acid S87A (MLH1^S87A^/PMS2; MLH1^S87A/S477A^/PMS2), showed very similar thermostability compared to the MutLα wt complex. In contrast, MLH1^S87A^/PMS2 as well as MLH1^S87A/S477A^/PMS2 showed impaired thermostability which manifests in a significantly flatter curve (Fig. 5A + C). Looking at the complexes after treatment with Calyculin and therefore hyperphosphorylation, the thermostability of the MutLα variants was very different (Fig. 5B + D). The lowest thermostability (with first derivative of the fluorescence intensity (FI) (Fi350nm/Fi330nm) = peak at 0.0035) was shown by the hyperphosphorylated MutLα wt and the MLH1^S477A^/PMS2 variant (first derivative (Fi350nm/Fi330nm) = peak at 0.0027). The highest thermostability after treatment with Calyculin was shown by MLH1^S456A^/PMS2 (first derivative (Fi350nm/Fi330nm) = peak at 0.0064) followed by MLH1^S446A^/PMS2 (first derivative (Fi350nm/Fi330nm) = peak at 0.0057) and MLH1^S87A^/PMS2 (first derivative (Fi350nm/Fi330nm) = peak at 0.0053) (Fig. 5B).

For the MutLα variants that carried two changes, all variants were found to have higher thermostability than the MutLα wt after treatment with Calyculin (Fig. 5D). The peak of the MutLα double variants were all on very similar levels: MLH1^S87A/S477A^/PMS2 where the first derivative (Fi350nm/Fi330nm) peak was at 0.0051, MLH1^S446A/S477A^/PMS2 where the first derivative (Fi350nm/Fi330nm) peak was at 0.0050 and MLH1^S456A/S477A^/ PMS2 where the first derivative (Fi350nm/Fi330nm) peak was at 0.0059 (Fig. 5D).

## The majority of the examined phosphorylation sites are located in the linker region of MLH1

**Figure 6:**
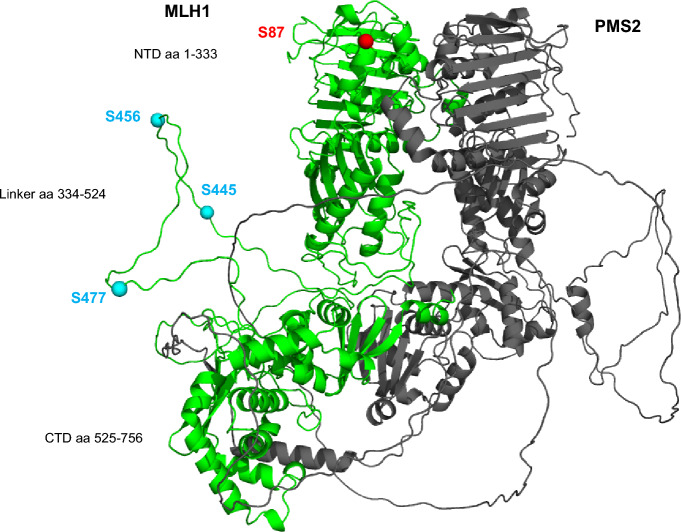
Potential structure of the p-MLH1^S87/S446/S456/S477^ / PMS2 heterodimer. The biologically active structural form of MLH1 (green) in its heterodimeric form with PMS2 (grey) is shown. The structure is a prediction based on AlphaFold2^23^. S87 is located in the ATPase domain (indicated as red ball) whereas S446, S456 and S477 of MLH1 are located in the flexible linker region and are indicated as light blue balls (shown on the left side). aa: amino acid; NTD: N-terminal domain; CTD: C-terminal domain.

In order to visualize the localization of S87, S446, S456 and S477 in silico, we used Pymol and modelled the biologically active structural form of dimeric MutLα (Fig. 6). While S87 (Fig. 6, red ball) is located in the N-terminal ATPase and MutS interacting domain of MLH1, all other phosphorylatable serines of MLH1 are localized in its flexible linker region (Fig. 6, light blue balls).

## Discussion

The human MMR is a highly complex mechanism for the maintenance of genetic information. Its functionality depends on diverse factors e.g. germline mutations^24^, promoter hypermethylation^25,26^ and posttranslational modification^14,27–29^. Recent mass spectrometry studies of our group showed that MLH1 is phosphorylatable at several sites, albeit with varying intensity (see supplementary data of^14^). In the present study, we investigated the effect of phosphorylation of MLH1 at amino acid position S87, S446, S456, and S477 in different recombinant MutLα variants and analyzed their protein expression and stability, DNA binding ability and MMR functionality in vitro. We were able to demonstrate for the first time that the ability of MutLα to bind to the DNA seems to be orchestrated via a phosphorylation status-dependent fine tuning and partial interplay between the N-terminal ATPase domain as well as serines located in the linker region of MLH1.

Our major finding is that S87, which is located in the N-terminal ATPase site of MLH1, is most essential for DNA binding of MutLα and that phosphorylation of S87 destroys this ability. The MLH1^S87A^/PMS2 variant, which is non-phosphorylatable at position S87, showed significantly enhanced binding to the DNA compared to MutLα wt. Interestingly, its DNA binding capacity was not influenced by the phosphorylation of all serine positions in the linker region. In contrast, the phosphorylated form of MLH1^S87A^/PMS2 showed the worst and most significantly reduced MMR activity compared to all other variants. Furthermore, we were able to detect that phosphorylation of S446 and S456 in the linker region of MutLα variants was enough to avoid the DNA binding as long as S87 as well as S477 were not phosphorylatable.

A general relevance of DNA binding of MutL for the MMR process has been demonstrated by Dortschmann et al*.* by using yeast Mlh1-Pms1 heterodimer^30^. Our observation that the linker region of MLH1 also seems to be important for DNA binding as well fits with Plys et al*.* who showed that the unstructured linker domains of MLH proteins of yeast provided distinct interactions with DNA during MMR^31^. The observation that DNA binding of MutLα seems to depend on amino acids of the N-terminus as well as partially on the linker region, is closely in line with previously published data for MutLγ, a MutL heterodimer which has been implicated in the processing of meiotic recombination intermediates into crossovers^32^. In their study, Bouuaert and Keeney mapped DNA contacts of MutLγ and showed that the DNA binding sites of MutLγ for single stranded DNA were located in the N-terminal ATPase domain as well as in the linker region^32^. Interestingly, the authors additionally demonstrated that most contacts with Holiday junction substrates were solely detectable in the linker region. Since DNA binding in our study was analyzed by using only one DNA substrate, we cannot give any information regarding the DNA binding ability to different substrates. However, this would be worthwhile to investigate more detailed in future studies and might give an explanation for the observation that phosphorylation of serines in the linker region differentially affects the DNA binding of MutLα variants in correlation to the phosphorylation status of S87. Another question is why single amino acid changes in the linker region show this very strong effects on DNA binding even in the unphosphorylated status of most used MutLα variants. We can only speculate about this and assume that the flexibility of the linker region of MutLα allows interactions within the own protein and that changes of single amino acids in the linker can significantly affect protein structure and function, which has been demonstrated for other proteins before^33^. Of course, we have to emphasize here that the DNA binding assay used in the current study was intended to focus solely on MutLα, therefore, no other proteins were included in this assay. The DNA binding behavior of MLH1 in combination with components of the MMR cannot be answered in the present case and has to be analyzed in detail by future experiments.

Concerning our detection of a phosphorylation-dependent regulation of the N-terminus of MLH1 in which the ATPase is located, one has to point out that the regulation of ATPases by phosphorylation is a common mechanism. A cAMP activated protein kinase phosphorylation site is e.g. responsible for regulating the pumping activity of Na^+^, K^+^- and H^+^, K^+^-ATPases, either by changing the cellular distribution of the ATPases or by directly altering their kinetic properties^34,35^. Positions of amino acids which are directly responsible for ATP binding in the ATPase region of MutLα have been already described for yeast^36^. Based on crystal structures of the N-terminal domain of yeast MutL, two conserved asparagine residues (position 35 in Mlh1 and position 65 in Pms1) have been shown as essential for ATP binding and two Glutamates (position 31 in Mlh1 and position 61 in Pms1) as important for ATP hydrolysis^36^. Since human and yeast MutLα are predominantly homologous, position S87 of human MLH1 is highly unlikely to be directly involved in ATP binding and phosphorylation at this position rather seems to have an indirect effect.

Considering the ATPase activity of MMR proteins, it is still not finally clarified in which step of the MMR process the respective ATPases are actually involved. It has been detected that, although the interaction of MutLα and MutSα requires ATP, the ATPase activity of MutLα is not essential for the formation of ternary complexes with MutSα, but required for downstream processes^37,38^. ATP hydrolysis by MutS and MutL is involved in the mismatch-dependent activation of MutL endonuclease activity^39^ and enables MutL to act as a switch to coordinate MMR^40^. Furthermore, it has been suggested that the ATPase activity of MutLα is regulated by conformational changes from an ADP bound open to an ATP bound condensed form, thus modulated by adenine nucleotides in the ATPase domain^41^. These changes have been identified to mediate the interaction of MutLα with other proteins in the MMR pathway and are of importance for the recognition of DNA mismatches by MutSα. As a consequence, MutLα is able to coordinate downstream events that lead to repair^41^ and the conformational status of MutLα also impacts its interaction with PCNA^42^ which is assumed to be necessary for strand discrimination^43^. Therefore, we hypothesize that the phosphorylation of MLH1 at position S87 might induce conformational changes at the catalytic interface of the N-terminus in the ATP binding region in a way that its ATP binding and ATPase function is suppressed, which might prevent the interaction with MutSα as well as the DNA binding and finally the MMR mechanism.

Our differential results concerning phosphorylation-dependent DNA binding and MMR functionality of MutLα lead us to the hypothesis that binding of MutLα to the DNA is regulated most dominantly by phosphorylation of S87 in the N-terminal ATPase domain while MMR activity is rather regulated by phosphorylation of positions in the linker region of MLH1. While the prevention of phosphorylation at position S87 increased DNA binding, which also remained in the case of phosphorylation of amino acids in the linker region, the phosphorylated form of this MLH1^S87A^/PMS2 variant showed the lowest MMR activity. In contrast, incomplete phosphorylatability of positions in the linker domain, especially in the presence of S477A, allowed MMR activity which is in line with our recently published data^14^ and underlines the importance of amino acid S477 for the regulation of the MMR. To our surprise, hyperphosphorylated variant MLH1^S446A^/PMS2 showed a significant reduction of MMR activity. The hypothesis that even minor changes in the linker region could affect protein structure and function, as mentioned above, might also explain this MMR result. However, this needs to be clarified by further experiments. 

The thermal stability data of the tested MutLα variants underline our observations of a phosphorylation-dependent regulation and showed that, overall, MutLα is less stable in the phosphorylated state than in the non-phosphorylated state. The stability of the complex is inversely correlated to the number of phosphorylated positions which seems to be in accordance with the loss of DNA binding and MMR function that we identified. Although these differences do not manifest themselves until a non-physiological temperature range is reached, they are nevertheless consistent with Becher et al*.* who analyzed protein thermal stability variation during the cell cycle and found that protein thermal stability serves as a proxy for enzyme activity, DNA binding, and complex formation^44^. Discussing a phosphorylation-dependent regulation of MutLα stability, it also seems appropriate to mention, that phosphorylation can promote or inhibit ubiquitination, which in turn can lead to proteasomal protein degradation among other consequences^45,46^. Thus, it is also conceivable that complete phosphorylation of MutLα is in the end required to degrade the protein complex and remove it from the MMR process.

Finally, it remains to be elucidated why phosphorylation of MLH1 was predominantly detected at position S477 in the mass spectrometry analysis, while all other serines were detected to a significantly lower proportion (S87 (6%), S446 (25%), S456 (5%), S477 (61%))^14^. We could imagine that other posttranslational modifications, e.g. ubiquitination^45,46^, the order of addition or removal of post translational modifications in the lifespan of the protein^47^, or the abundance of special kinases and phosphatases^48,49^ might significantly impact the phosphorylatability of the investigated positions.

Altogether, one might speculate that the complete detachment of the MutLα complex from the DNA is a process that occurs rather infrequently, whereas the direct acting in the context of MMR is more precisely regulated. Descriptions of the clamp of MutS, which actually slides along the DNA during the entire replication process and only locks at a certain position when an error occurs, would fit this assumption^50^. However, this hypothesis has to be investigated in more detail in future.

## Conclusions

**Figure 7:**
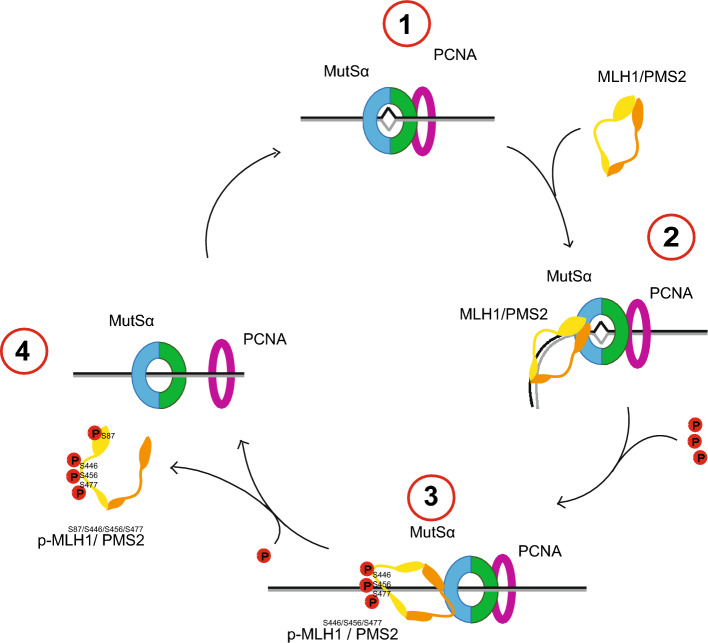
Model of phosphorylation-dependent binding, activity and dissociation of MutLα during the MMR process. 1. Initiation of the MMR after binding of MutSα. 2. Starting process and performance of the MMR after MutLα binding and activity. 3. Stop of the MMR process after phosphorylation of MLH1 at positions S446, S456 and S477. 4. Separation of MutLα from the DNA after phosphorylation of MLH1 at positon S87.

In summary, we hypothesize a phosphorylation-dependent fine tuning of the MutLα complex during the MMR process. While the phosphorylation of serines located in the linker region seems to be responsible for interrupting the ability of MutLα to participate in the MMR process, the phosphorylation of S87, located in the N-terminal ATPase domain, might finally cause the dissociation of MutLα from the MMR complex (Fig. 7).

## Supplementary Information

Supplementary Information 1.

Supplementary Information 2.

## Data Availability

All datasets used and/or analysed during the current study are shown in the supplementary data. For any other additional data desired on reasonable request, please contact the corresponding author.
