# Supplementary material for: Key role of phosphorylation sites in ATPase domain and Linker region of MLH1 for DNA binding and functionality of MutLα
Source: Sci Rep. 2023 Aug 2;13:12503. doi: 10.1038/s41598-023-39750-x (PMC10397344; doi:10.1038/s41598-023-39750-x)
Supplement: Supplementary file 1 — Supplementary Information 1. [file 41598_2023_39750_MOESM1_ESM.pdf]

Figure S1

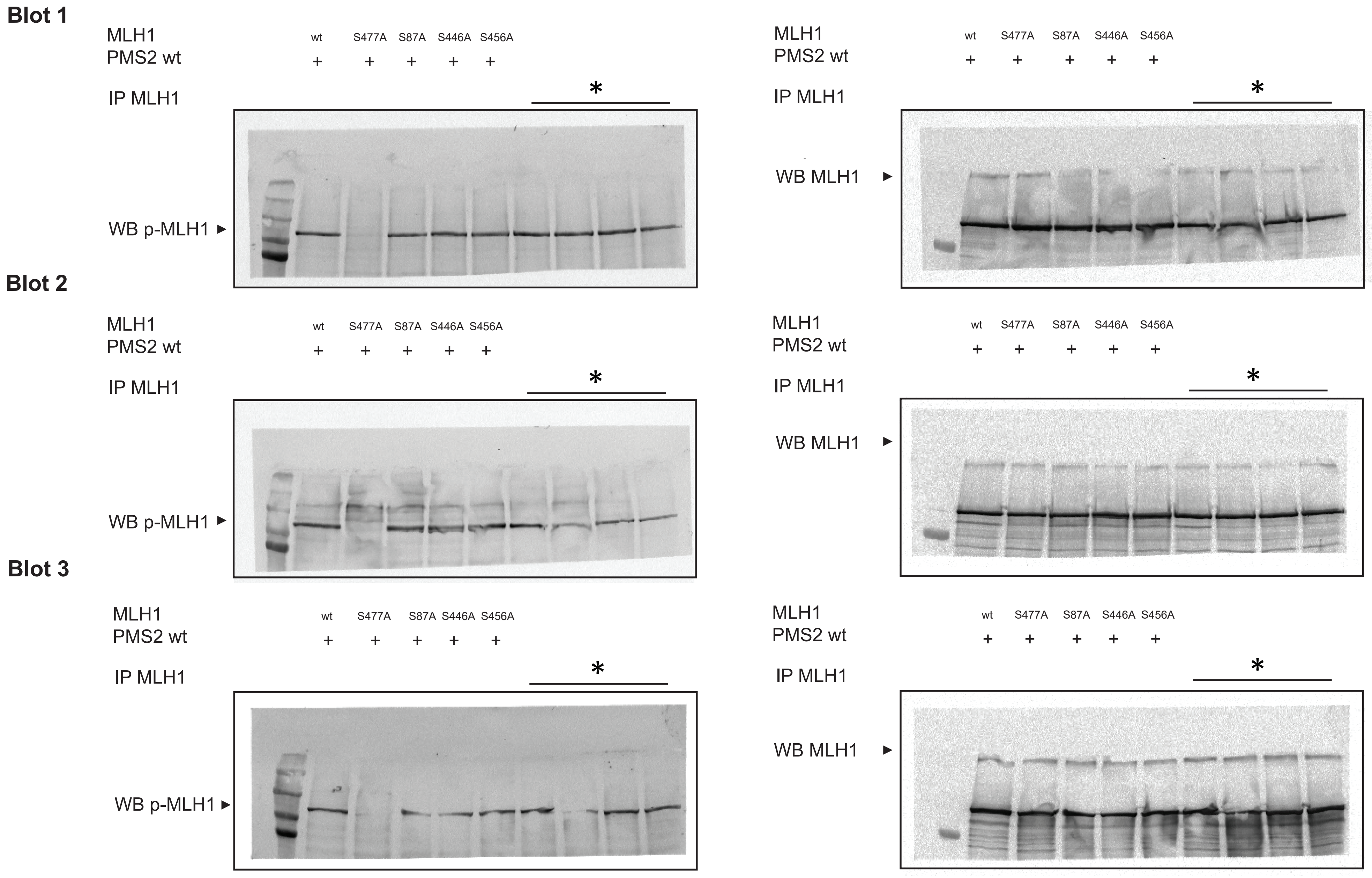

*Data not used.

Figure S2

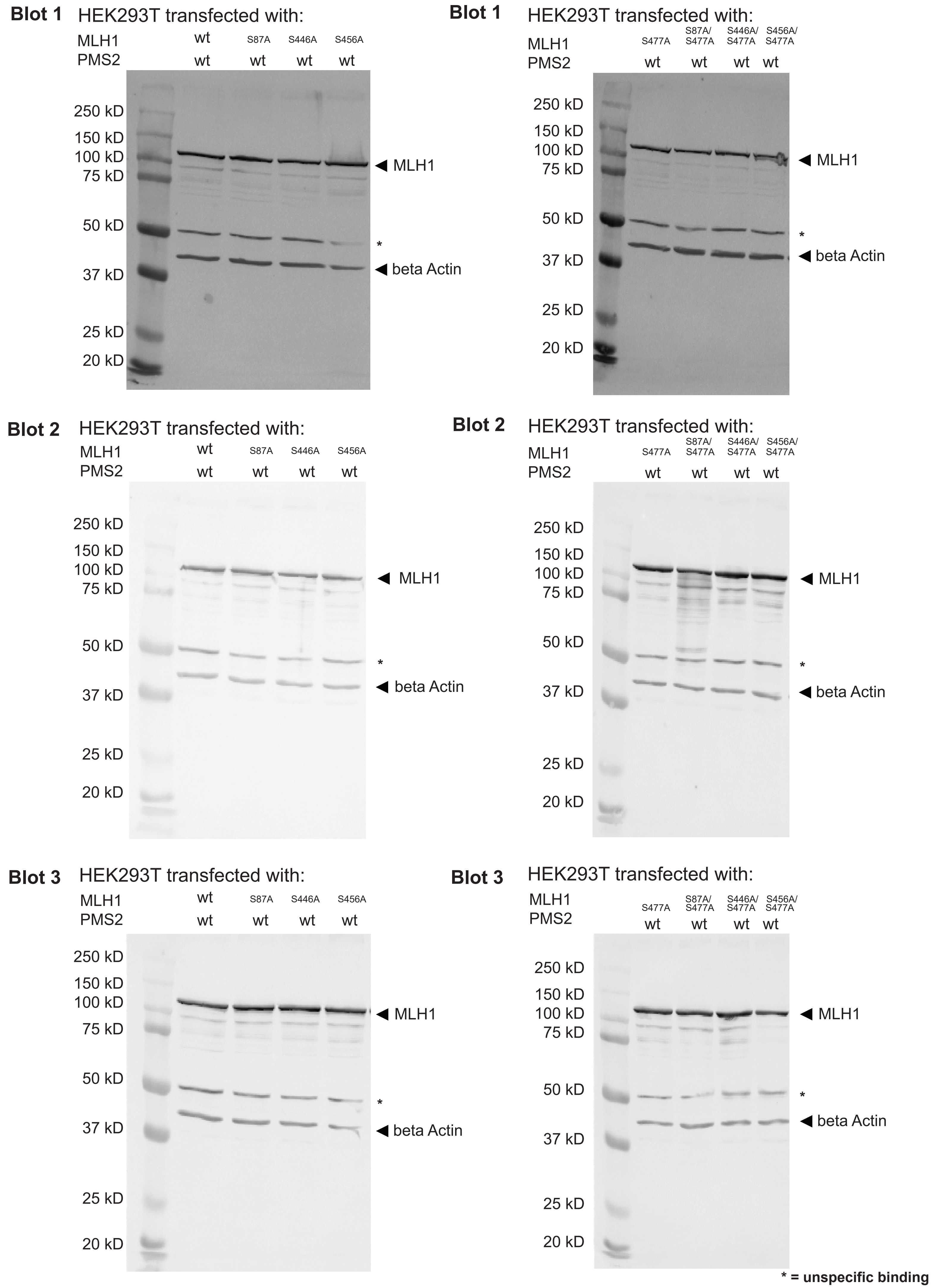

* = unspecific binding

Figure S3

A

MutLα wt

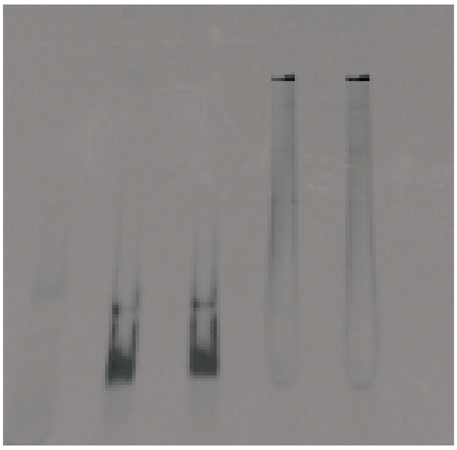

Marker	+	-	-	-	-
DNA substrate	-	+	+	+	+
Sepharose	-	-	+	-	-
Recombinant protein	-	-	-	+	-
Sepharose-bound protein	-	-	-	-	+

B

MutLα wt

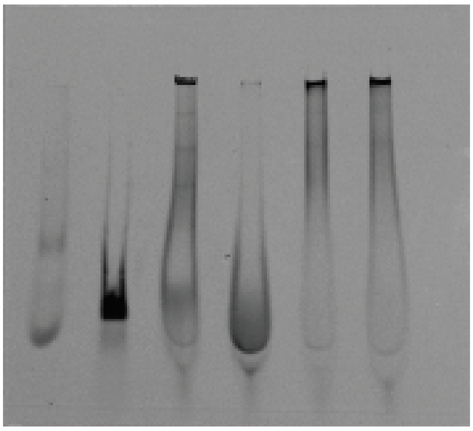

Marker	+	-	-	-	-	-
DNA substrate	-	+	+	+	+	+
Sepharose-bound protein	-	-	+	+	+	+
37°C	-	-	+	-	+	-
CIP	-	-	+	-	-	-
Calyculin	-	-	-	+	-	-

Figure S4

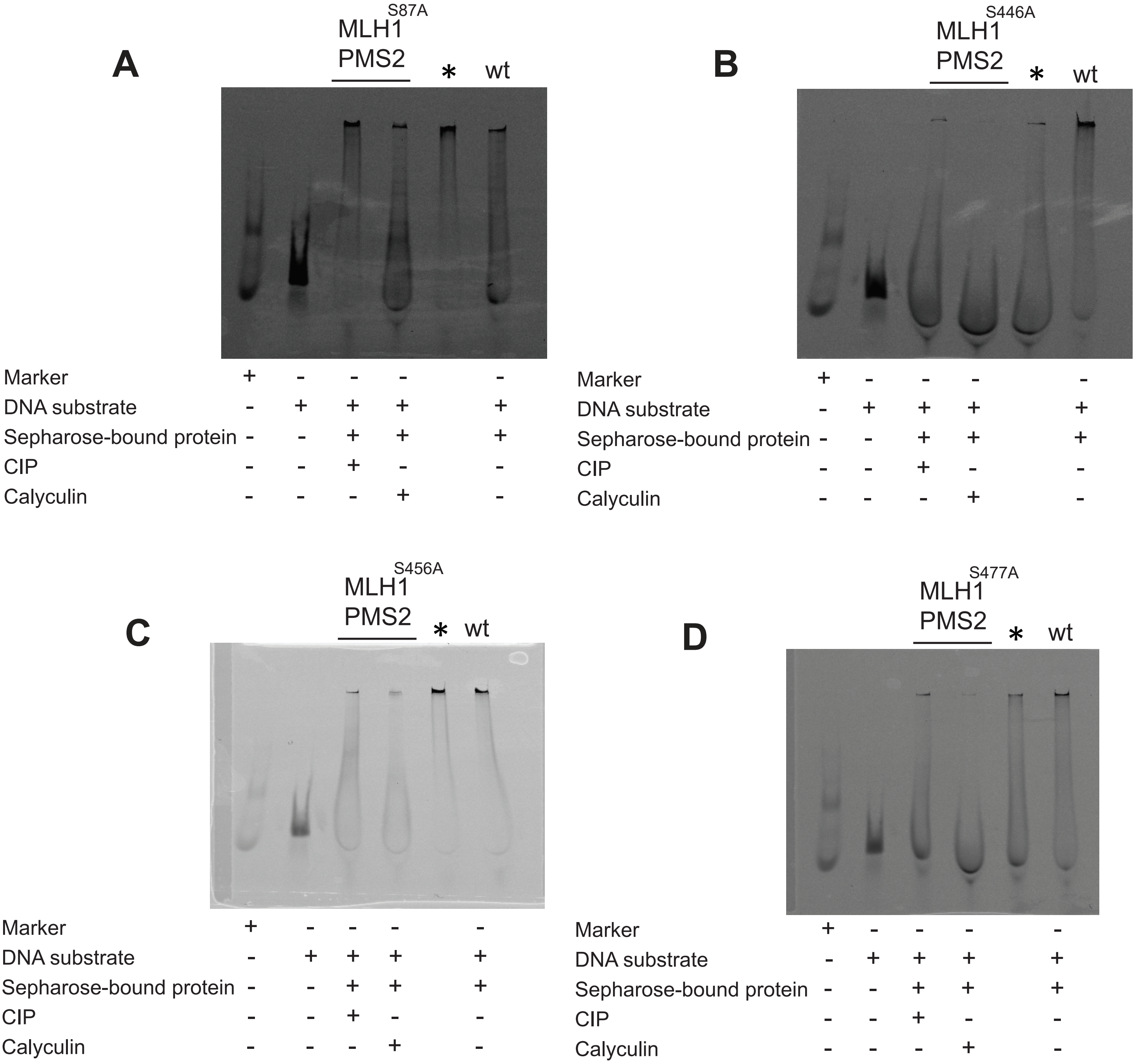

*Data not used.

Figure S4

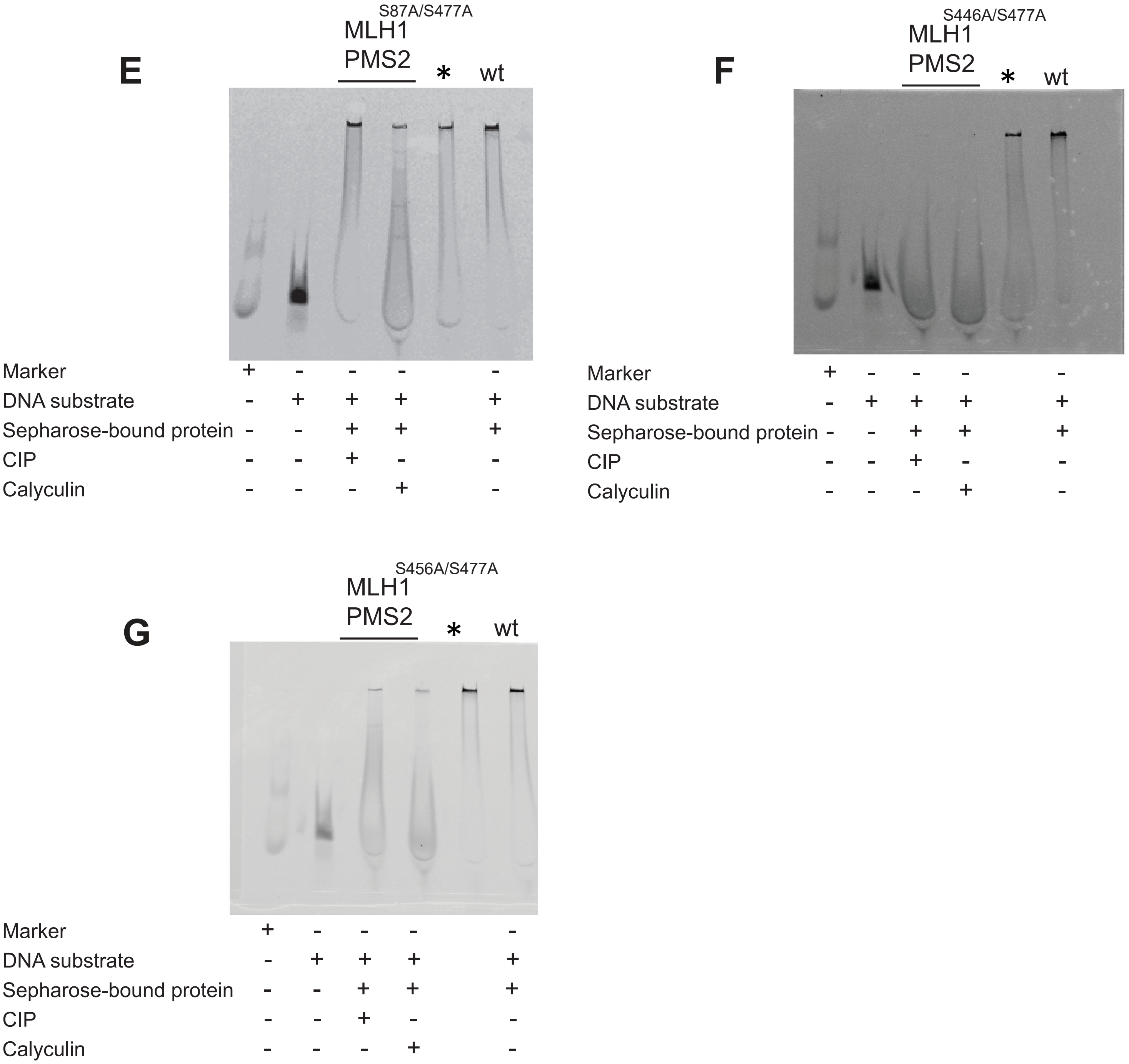

* Data not used.

Figure S5A

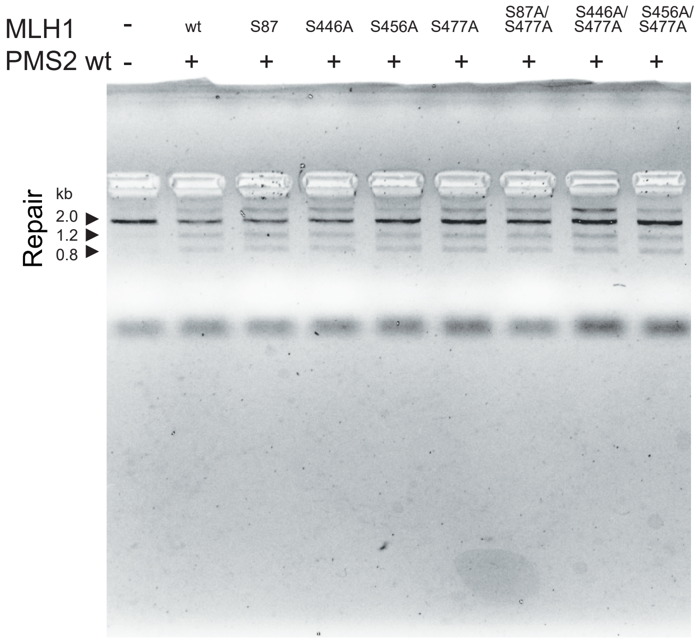

MMR activity									
absolute	4.75	39.72	39.26	40.36	28.87	32.62	19.42	29.25	29.84
relative	11.97	100.00	98.85	101.63	72.70	82.14	48.90	73.64	75.13

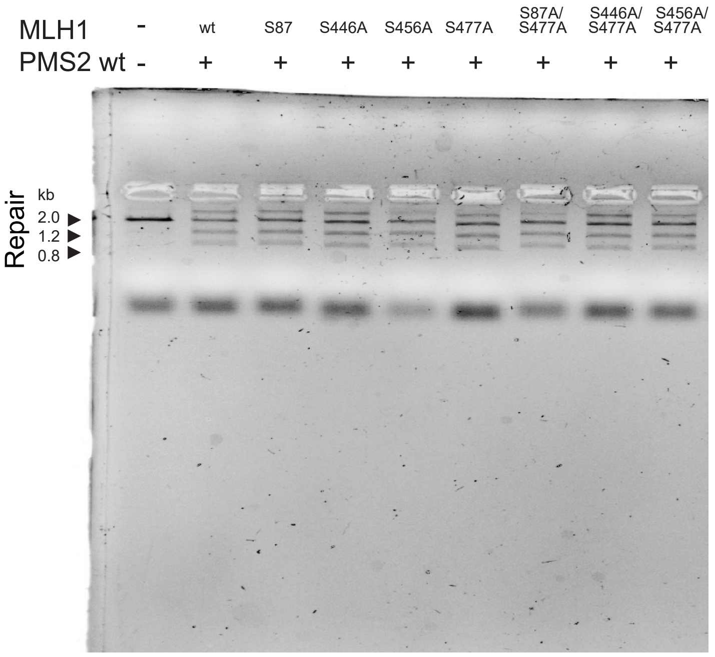

MMR activity									
absolute	1.94	45.72	51.43	57.24	58.72	47.82	43.13	58.07	51.32
relative	4.24	100.00	112.47	125.17	128.42	104.58	94.33	126.99	112.23

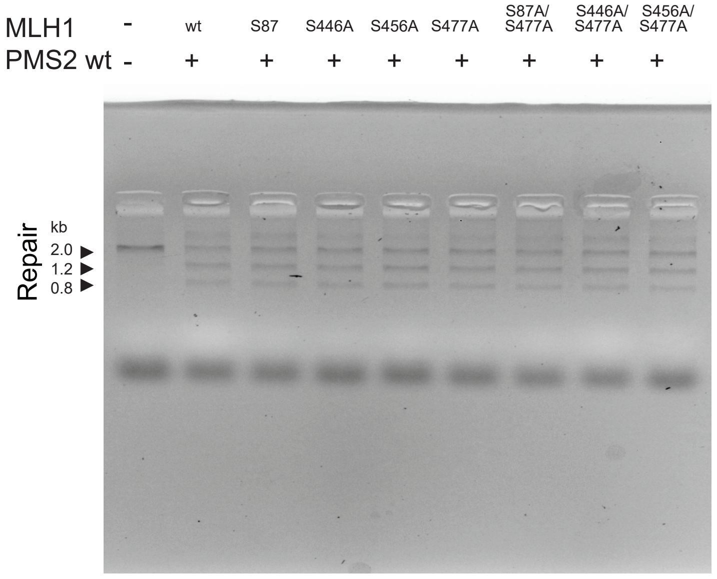

MMR activity									
absolute	3.30	83.20	45.27	73.12	55.67	43.58	75.42	66.44	64.97
relative	3.02	100.00	54.86	83.60	67.31	52.34	90.57	81.14	77.18

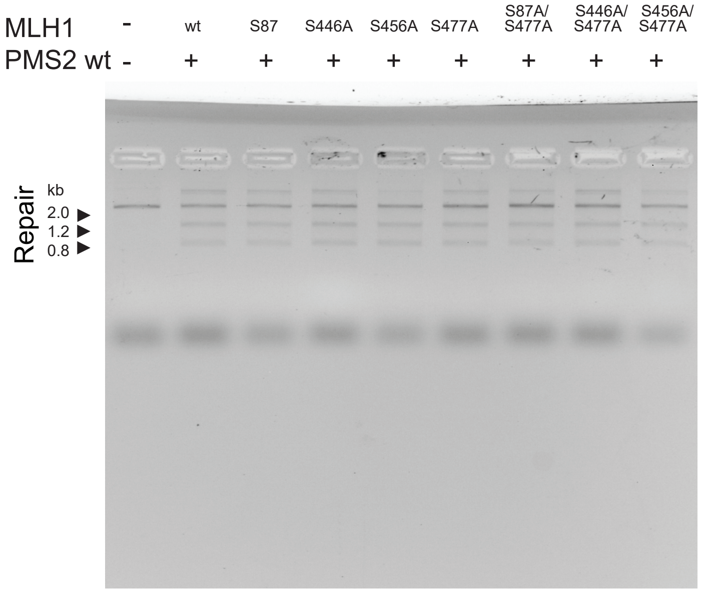

MMR activity									
absolute	1.89	39.09	34.07	33.44	35.69	31.69	24.17	33.04	34.78
relative	4.82	100.00	87.15	85.53	91.30	81.07	61.83	84.52	88.98

Figure S5B

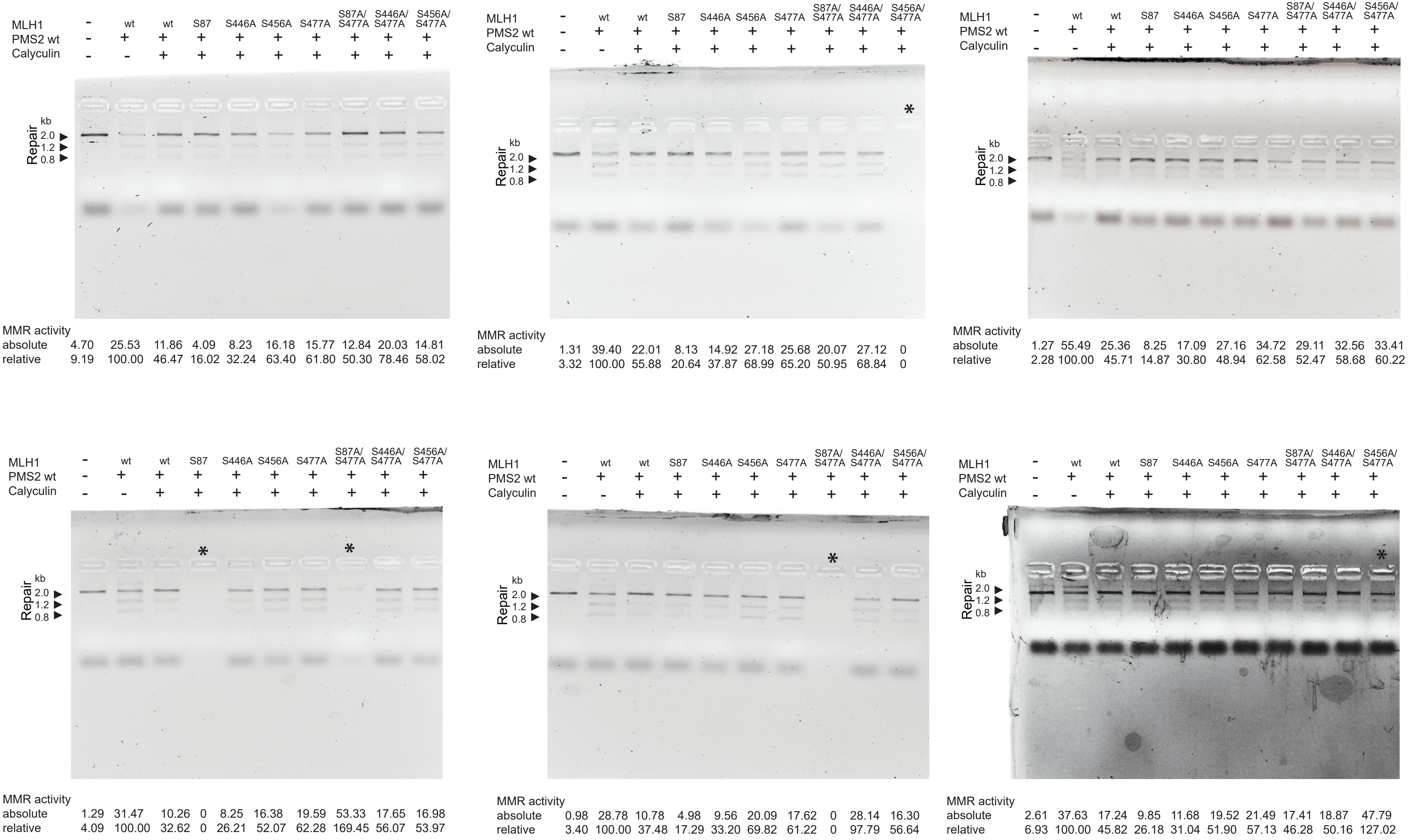

* Data not used for calculation.

Figure S6

A

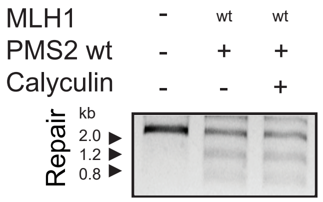
MLH1 - wt wt
PMS2 wt - + +
Calyculin - - +
Repair
kb
2.0
1.2
0.8

B

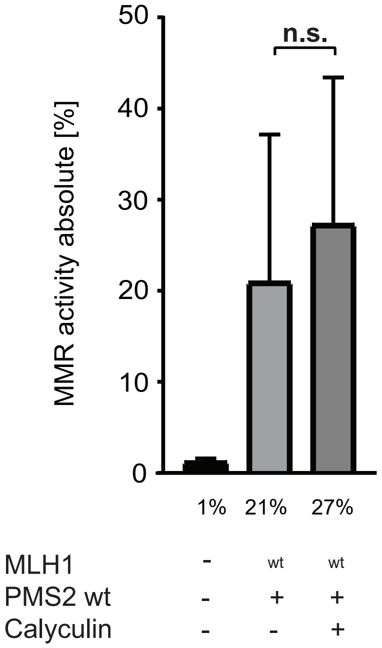
MMR activity absolute [%]
0
10
20
30
40
50
n.s.
1%
21%
27%
MLH1 - wt wt
PMS2 wt - + +
Calyculin - - +
