# Supplementary material for: Key role of phosphorylation sites in ATPase domain and Linker region of MLH1 for DNA binding and functionality of MutLα
Source: Sci Rep. 2023 Aug 2;13:12503. doi: 10.1038/s41598-023-39750-x (PMC10397344; doi:10.1038/s41598-023-39750-x)
Supplement: Supplementary file 2 — Supplementary Information 2. [file 41598_2023_39750_MOESM2_ESM.pdf]

## Figure legends
## Supplementary figures

**Figure S1: Western blots corresponding to Figure 1A showing full-length/uncropped blots and all bands and molecular weight markers.** Using immunoprecipitated MutL$\alpha$ **(A)** p-MLH1$^{S477}$ was detected with a specific antibody which recognizes the phospho-S477-motif of MLH1 and **(B)** the whole amount of MLH1 was determined with a specific anti-MLH1 in MutL$\alpha$ wt as well as in MLH1$^{S477A}$/PMS2, MLH1$^{S87A}$/PMS2, MLH1$^{S446A}$/PMS2, MLH1$^{S456A}$/PMS2 variant overexpressing HEK293T cells. Nitrocellulose membranes on which immunoprecipitated proteins were blotted were cut between 50 and 75kDa, only the upper part of the membrane was incubated with antibody. This prevented hybridisation of the antibody of interest to the antibody used for precipitation. Shown are the three Western blots which were used for quantification of the expression levels of p-MLH1$^{S477}$ (Figure 1B). p-MLH1$^{S477}$ was well detectable in all protein extracts despite in the extract of HEK293T cells overexpressing the nonphosphorylatable MLH1$^{S477A}$/PMS2 variant while MLH1 was detectable in all cellular extracts.

**Figure S2: Western blots corresponding to Figure 2A showing full-length/uncropped blots and all bands and molecular weight markers.** HEK293T cells were cotransfected with pEBG-2T/MLH1 or different pEBG-2T/MLH1 variants and pcDNA3.1+/PMS2. Protein expression of MLH1 was analyzed via Western blotting, beta Actin expression served as a control. The expression levels of all MLH1 variants were similar compared to MLH1 wt.

**Figure S3: Non-denaturing polyacrylamide gels performed for EMSA control experiments.** EMSA was carried out in 4% non-denaturing polyacrylamide gels. **(A)** The quality of sample-binding was determined by comparing the DNA binding capacity of 15 µg purified GST-tagged MutL$\alpha$ wt with the ability of 10 µl of Glutathione-sepharose-bound MutL$\alpha$ wt to bind to the DNA substrate. As negative control, the DNA substrate or pure sepharose was run without other supplements. **(B)** Dephosphorylated Glutathione-sepharose-bound MutL$\alpha$ wt (treated with CIP) and hyperphosphorylated MutL$\alpha$ wt (generated by Calyculin treatment) were analyzed and compared to untreated samples.
The quantity of DNA-bound protein was the same for purified GST-tagged MutL$\alpha$ in comparison to Glutathione-sepharose-bound MutL$\alpha$. Furthermore, dephosphorylated MutL$\alpha$ was able to bind well to DNA while hyperphosphorylation MutL$\alpha$ showed significantly reduced DNA binding ability.

**Figure S4: Non-denaturing polyacrylamide gels used for the generation of Figure 3.** EMSA was carried out in 4% non-denaturing polyacrylamide gels using Glutathione-sepharose-bound protein samples. Representative pictures show the amount of DNA-bound **(A)** MLH1$^{S87A}$/PMS2, **(B)** MLH1$^{S446A}$/PMS2, **(C)** MLH1$^{S456A}$/PMS2, **(D)** MLH1$^{S477A}$/PMS2, **(E)** MLH1$^{S87A/S477A}$/PMS2, **(F)** MLH1$^{S446A/S477A}$/PMS2, and **(G)** MLH1$^{S456A/S477A}$/PMS2 variant. Untreated Glutathione-sepharose MutL$\alpha$ wt complex served as positive control in all experiments. DNA substrate without other supplements was used as negative control. In parallel, a calculation of the exact amount of DNA-bound proteins was performed via Western blotting. Finally, the DNA-bound amount of MutL$\alpha$ wt was quantified, set to 100% and the amount of DNA-bound MutL$\alpha$ variants was correlated, respectively (Figure 3 A-H). All experiments were performed at least three times. * Data not used.

**Figure S5. Agarose-gels used for calculation of MMR activity data shown in Figure 4.** Shown are all agarose gels performed to analyze the MMR activities of **(A)** unphosphorylated and **(B)** hyperphosphorylated (generated by Calyculin treatment) MutL$\alpha$ variants and used to calculate the average values shown in Figure 4. As described in detail in the method section, three bands are detectable after running the protocol: a 2.0 kb band corresponding to singly cut (uncorrected and in excess added) DNA substrate, a 1.2 kb and a 0.8 kb band caused by the successfully corrected restriction site. The band intensities were quantified using Image Lab version 3.0 (Bio-Rad) and the absolute repair efficiency (e) was calculated as: (e) = intensity of bands of repaired substrate/intensity of all bands of substrate.

Thereafter, the relative MMR repair efficiency $(e)_{relative}$ was calculated and the (e) of MutL$\alpha$ variants was analyzed in direct comparison with MutL$\alpha$ wt and calculated in parallel as $(e)_{relative} = (e)_{variant}/(e)_{wild\text{-}type} \times 100$. Calculated absolute and relative MMR activity values are shown under each agarose gel. n=4-6; * Data were not taken into account for the calculation of the average values.

**Figure S6: Examination of the influence of separate Calyculin supplementation on the functionality of the MMR assay.** As mentioned in the manuscript, protein extracts of Calyculin treated cells were used to analyze the effects of phosphorylation. To exclude a potentially existing influence of Calyculin, an MMR assay was performed by incubating the DNA substrate with protein extracts of untreated GST-labeled MutL$\alpha$ wt and separately adding the calculated small amount of Calyculin, which was included in the MMR assay in case of using protein extracts of Calyculin treated cells. In parallel the MMR activity of untreated GST-labeled MutL$\alpha$ wt without a separate addition of Calyculin was determined. Shown are **(A)** a representative MMR assay and **(B)** the quantification of the MMR activity of the tested samples (n=3). There was no significant difference in MMR function detectable comparing the MMR function of untreated GST-tagged MutL$\alpha$ wt with those where Calyculin was added separately to the assay.
